# Five-Year Outcomes of Bariatric Surgery vs. Conservative Weight Management in People with HIV: A Single-Center Tertiary Care Experience

**DOI:** 10.1007/s11695-024-07443-7

**Published:** 2024-08-27

**Authors:** Matyas Fehervari, Anuja T. Mitra, Narek Sargsyan, Nuala Davison, Madeleine Turner, Evangelos Efthimiou, Haris Khwaja, Naim Fakih-Gomez, Gianluca Bonanomi

**Affiliations:** 1 Department of Bariatric Surgery, Chelsea and Westminster Hospital, London, UK; 2 Department of Surgery and Cancer, Imperial College London, London, UK; 3 Jaber Al Ahmad Hospital, Kuwait City, Kuwait

**Keywords:** HIV, Bariatric surgery, Obesity

## Abstract

**Introduction:**

Individuals with human immunodeficiency virus (HIV) infection now have life expectancies similar to non-infected people but face increased obesity prevalence. The long-term effects of bariatric surgery (BS) and conservative weight therapy (CWT) in patients living with HIV (PLWH) remain unexplored.

**Methods:**

A retrospective review (2012–2018) at a Tertiary Centre for Bariatric Surgery and National Centre for HIV care examined the outcomes of BS and CWT. Parameters evaluated included weight loss and HIV metrics such as viral load and CD4 count.

**Results:**

The study included 24 chronic HIV patients, with 10 undergoing BS (5 laparoscopic adjustable gastric banding (LAGB), 3 laparoscopic sleeve gastrectomy (LSG), 2 Roux-en-Y gastric bypass (LRYGB) and 14 in CWT. The BS group showed significant BMI reduction (− 7.07, − 6.55, − 7.81 kg/m^2^ at 1, 3, and 5 years). The CWT group’s BMI reduction was non-significant. The BS group’s %TWL was 16%, 17.8%, and 15% at 1, 3, and 5 years, respectively; however, stapled procedures were more effective, at 1 year, %TWL was 17% LSG and 25% RYGB, at 3 years, 23% LSG, 30% RYGB and at 5 years 21% with LSG and 28% with RYGB. HIV outcomes remained stable with undetectable viral loads in the BS group.

**Discussion:**

BS appears to be a safe and effective medium-term treatment for obesity in PLWH, providing significant weight loss whilst maintaining the efficacy of HIV treatments. Although CWT has shown modest benefits, the outcomes from BS indicate that it could be a preferable option for managing obesity in PLWH based on this limited dataset.

**Graphical Abstract:**

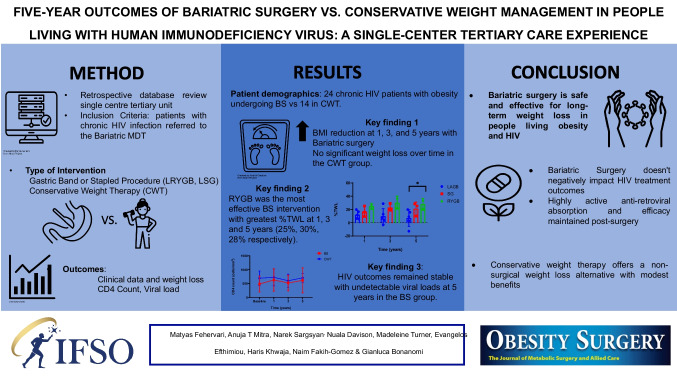

## Introduction

Human immunodeficiency virus (HIV) infection in adults is now recognized as a chronic disease, with life expectancies approaching those of non-infected individuals [1]. Once viewed as a disease associated with cachexia and slimming, the successful management of HIV has seen the prevalence of obesity in people living with HIV (PLWH) greatly increase, with the trajectory of obesity being mirrored in infected versus non-infected populations [2, 3]. Obesity is recognised as a medical disease in its own entity and is associated with a significantly increased risk of cardiovascular disease, type 2 diabetes, and other obesity-related complications. It is known to have a significant detrimental impact on the overall health and well-being of PLWH [1, 4–8], therefore weight management has started to be considered as an important component of the management in PLWH. Bariatric surgery has emerged as the most effective treatment option for obesity, leading to substantial and sustained weight loss, resolution of obesity-related comorbidities, improved life expectancy and quality of life [9–12]. The published literature base surrounding bariatric surgery and HIV is expanding with a variety of studies and case series investigating the outcomes of BS in PLWH. Fysekidis et al. (2015) presented a case series focusing on sleeve gastrectomy in HIV patients with morbid obesity, demonstrating favourable results in terms of weight loss, comorbidity evolution, and immune function at 20 months [13]. Outcomes from a tertiary bariatric surgical centre in France published their experience, reporting that bariatric surgery is safe and feasible in individuals with chronic HIV infection with maintenance of viral suppression loads and CD4 counts [14]. Kassir et al. in their letter to the editor summarised the available data on BS in HIV and provided insights into surgical considerations and postoperative outcomes suggesting that laparoscopic sleeve gastrectomy (LSG) appears to be a safer option than other bariatric surgical interventions [15]. However, a systematic literature review and meta-analysis involving 58 patients with chronic HIV infection and obesity demonstrated that the most common procedure being undertaken in HIV-infected individuals was RYGB (25 cases) followed by LSG (22 cases). Based on the available evidence, the review concluded that bariatric surgery is deemed safe in HIV-infected individuals and does not exert any additional adverse impact on the progression of HIV disease in the short term. Moreover, the analysis revealed no significant differences in HIV-related outcomes between sleeve gastrectomy (SG) and Roux-en-Y gastric bypass (RYGB) procedures [16].

Overall, the available data highlights the potential benefits of bariatric surgery, including substantial weight loss, improvement in metabolic parameters, and resolution of obesity-related comorbidities in PLWH. Despite its proven efficacy, bariatric surgery remains a relatively underutilized intervention in PLWH. Whilst there is moderate evidence on the short-term outcomes and safety of bariatric surgery in this population, there is limited medium and long-term data. The aim of this study was to evaluate the long-term weight specific and general outcomes of bariatric surgery in PLWH from a tertiary referral centre.

## Methods

A retrospective review was conducted on a prospectively collected local database comprising of consecutive patients referred to a Tertiary Centre for Bariatric Surgery and National Centre for HIV care between 2012 and 2018. All patients referred, followed a protocolised bariatric management pathway and were discussed during multidisciplinary team meetings. Outcomes reported in the database included comprehensive clinical and demographic data, surgical metadata, postoperative complications, pre-operative and postoperative CD4 count antiviral load, changes to antiretroviral therapy, weight loss outcomes and morbidity and mortality. The range of bariatric surgical interventions utilised included laparoscopic adjustable gastric banding [LAGB], laparoscopic sleeve gastrectomy [LSG], and laparoscopic gastric bypass [RYGB]). Non-surgical weight loss strategies involved enrolment in a Supervised Conservative Weight Therapy (CWT) programme. This programme comprised group sessions led by psychologists and dietitians, supplemented by frequent one-on-one consultations with a dietitian and psychologist. Additionally, this patient group participated in regular physician reviews and, for some patients, sessions with a physiotherapist (Appendix 1). The data collection was approved by the Research and Development Office at our Institution (Reference number: PCD906).

Assessment of weight loss and weight regain included calculations of the percentage of total weight loss (TWL%), percentage of excess weight loss (EWL%), and body mass index (BMI), following the methodology described by Brethauer et al. [17]. Follow-ups of HIV parameters were undertaken by the HIV team which included viral load and CD4 count assessment. Weight outcomes for CWT and BS patients were extracted at 1 and 3 years and those undergoing BS were further followed up for 5 years. HIV outcomes were followed up at 1, 3 and 5 years for all patients.

### Statistical Analysis

Statistical analysis was performed with Prism for Windows 5.01 (GraphPad Software, San Diego, CA, USA) and SPSS for Mac OSX 21.0.0 (SPSS Inc., Chicago, IL, USA) statistical software products. As many of the variables had non-Gaussian distributions, we used nonparametric tests for the analysis. We used the Mann–Whitney’s *U* test to compare two independent groups and Kruskal–Wallis one-way ANOVA for comparing multiple groups. Contingency tables were analysed by Fisher Exact Test and Chi Square Test. Statistical analyses were performed two-tailed and *p* < 0.05 was considered as significant. Values presented in the text are median, minimum and maximum values in brackets unless otherwise stated.

## Results

There were 24 patients referred to the Bariatric MDT with chronic HIV infection during the period of this study. Of those, 10 underwent bariatric surgery whilst 14 patients were placed on a CWT programme. The decision for the intervention assignment was made by several factors influencing the bariatric MDT. In 6 cases, patients had a BMI below 40 kg/m^2^ without any significant obesity related comorbidities, hence they did not qualify for BS based on the UK National Institute of Clinical Excellence guidelines. Two individuals were experiencing challenges related to their mental health and two individuals reported active substance use issues, which currently preclude them from qualifying for bariatric surgery. Three individuals declined surgical intervention and one case was deemed too high risk due to being classified as having BMI over 50 kg/m^2^ with concomitant cardiac issues.

Patients undergoing BS were significantly younger (43.5 versus 54.5 years, *p* = 0.006) with higher baseline BMI than those on CWT programmes (BMI 48.7 kg/m^2^ versus 41.3 kg/m^2^, *p* = 0.05). Full baseline patient characteristics are presented in Table 1.

**Table 1:** Baseline demographic data and comorbidities of patients living with HIV and obesity who were referred to the Bariatric service between 2006 and 2012

	Bariatric surgery	Conservative weight therapy	Difference (*p* value)
Total	10	14	
Age (years)	41.8 (37.8–46)	50.67 (44.5–57.3)	*p* = 0.017
Gender (female)	60% (6)	50% (7)	0.69
Type of intervention	LAGB: 5 LSG:3 RYGB:2	Supervised diet	
Initial BMI (kg/m^2^)	48.7 (36.7–57.9)	41.3 (32.8–52.2)	*p* = 0.05
Initial weight (kg)	146.4(106–182.8)	120.5 (90.9–159)	*p* = 0.08
Current smoker	30% (3)	14% (2)	0.52
T2DM	50% (5)	50% (7)	1
CV disease	20% (2)	29% (4)	1
OSA	44% (4)	57% (8)	0.67
Dyslipidaemia	40% (4)	50% (7)	0.92
Fatty liver disease	30% (3)	36% (5)	1
Hypertension	50% (5)	57% (8)	0.92
Asthma	30% (3)	21% (3)	0.47
GORD	40% (4)	43% (6)	0.34
Depression	60% (6)	50% (7)	0.34

### Weight Loss Outcomes

Ten patients underwent weight loss surgery during the study period including which consisted of 5 LAGBs, 3 LSGs and 2 LRYGBs. The baseline BMI was greater in the BS group in comparison to the CWT group, (BMI 48.7 kg/m^2^ versus 41.3 kg/m^2^, *p* = 0.05).

**Fig. 1:**
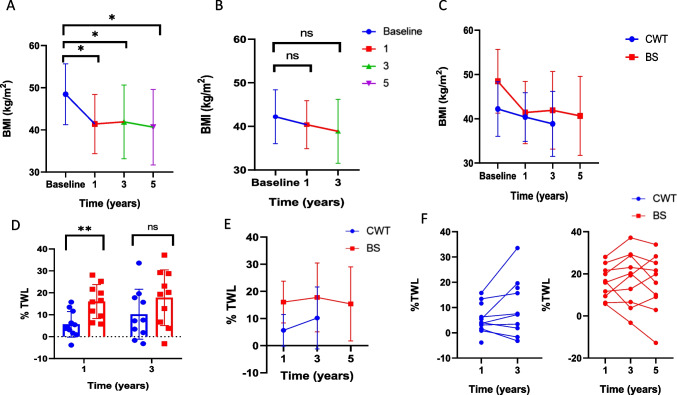
Change in body mass index (BMI kg/m^2^) in patients undergoing (**A**) bariatric surgery, (**B**) conservative weight programmes (CWT) at 1, 3 and 5 years, (**C**) BMI trends over time in both groups, (**D**) %total weight loss (%TWL) comparing BS and CWT groups at 1 and 3 years, (**E**) %TWL trends over time in both groups and (**F**) individual %TWL scores in patients in BS and CWT groups. Results are reported as mean and standard error of the mean. Significance is highlighted as * for *p* < 0.05, ** *p* < 0.001

Patients undergoing BS had significant reduction in their BMI at 1-, 3- and 5-year, BMI − 7.07 kg/m^2^ (*p* = 0.02), − 6.55 kg/m^2^ (*p* = 0.04) and − 7.81 kg/m^2^ (*p* = 0.01) respectively (Fig. 1a). In comparison, there was a non-significant reduction in BMI in the CWT group at 1 and 3 years, mean BMI 40.39 at 1 year (*p* = 0.71) and 38.86 kg/m^2^ (*p* = 0.36) at 3 years (Fig. 1b). A comparison to BMI trends in both groups is presented in Fig. 1c.

In terms of TWL, there was a significantly greater %TWL at 1 year in patients undergoing BS in comparison to CWT, 16% versus 5.6%, *p* = 0.002. At 3 years, patients achieved greater overall %TWL in both groups, mean %TWL BS 17.8% versus 10.2%, *p* = 0.09) (Fig. 1d). The %TWL in those undergoing BS was 16%, 17.8% and 15% at 1, 3, and 5 years respectively, reaching its peak at 3 years, demonstrating stable and sustained weight loss. In comparison with those undergoing CWT, there was modest %TWL at 1 year of 5.6% which increased to 10% at 3 years (Fig. 1e). Individual %TWL data at 1 and 3 years is presented in Fig. 1f.

**Fig. 2:**
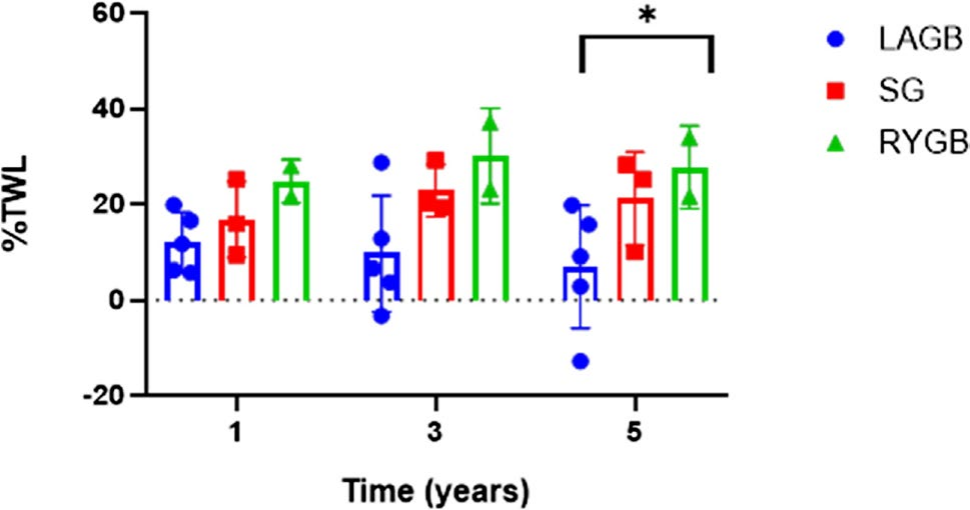
Percentage Total Weight Loss (%TWL) following laparoscopic gastric banding (LAGB) *n* = 5, laparoscopic sleeve gastrectomy (LSG) *n* = 3 and Roux-en-Ygastric bypass (RYGB) *n* = 2 at 1, 3 and 5 years in patients living with HIV following surgery. Results are reported as mean and standard error of the mean, significance is highlighted as * = *p* < 0.05

On subgroup analysis based on BS intervention, there was a significantly greater weight loss with stapled procedures than LAGB, with RYGB being the most effective, Fig. 2. At 1 year, %TWL was 12% LAGB, 17% LSG and 25% RYGB. This trend continued at 3 and 5 years, %TWL 8% LAGB, 23% LSG, 30% RYGB and at 5 years, 7% TWL LAGB, 21% with LSG and 28% with RYGB.

### Perioperative Complications

One LAGB was removed 2 years after insertion due to infection. This patient achieved 5.7%TWL after 1 year however regained some weight prior to removal (− 3.2%TWL). One patient returned to theatres for evacuation of a port site heamatoma.

There was no direct mortality related to the surgery but 1 mortality in the CWT group from myocardial infarction in the patient who was deemed high risk by the bariatric MDT and was considered unfit to undergo surgical intervention.

### HIV Outcomes

All patients were on active treatment with ant-retrovirals throughout the study overseen by the specialist HIV team at Chelsea and Westminster Hospital. There were no reports of non-compliance in either group.

### Viral Load

At baseline, 90% of patients in the BS group and 80% in the CWT group had an undetectable viral load (less than 50 copies per millilitre of blood). All patients had undetectable viral loads in the BS group at follow-up at 1, 3, and 5 years. The viral load in patients on CWT programme demonstrated more fluctuation, with 93% of patients having an undetectable load at 1 year on the programme however dropping to 80% and 85% at 3 and 5 years respectively, Table 2.

**Table 2:** Proportion of patients with an Undetectable viral load. Proportion of patients given as percentages (%) with an undetectable viral load (< 50 copies per millilitre) on follow-up at 1-, 3- and 5-year form either bariatric surgery (BS) or on a conservative weight management programme (CWT)

	Bariatric surgery	Conservative weight therapy
Baseline % (*n*)	90% (9)	80%
Viral load at 1 year	100	93%
Viral load at 3 years	100	80
Viral load at 5 years	100	85

### CD4 Count

**Fig. 3:**
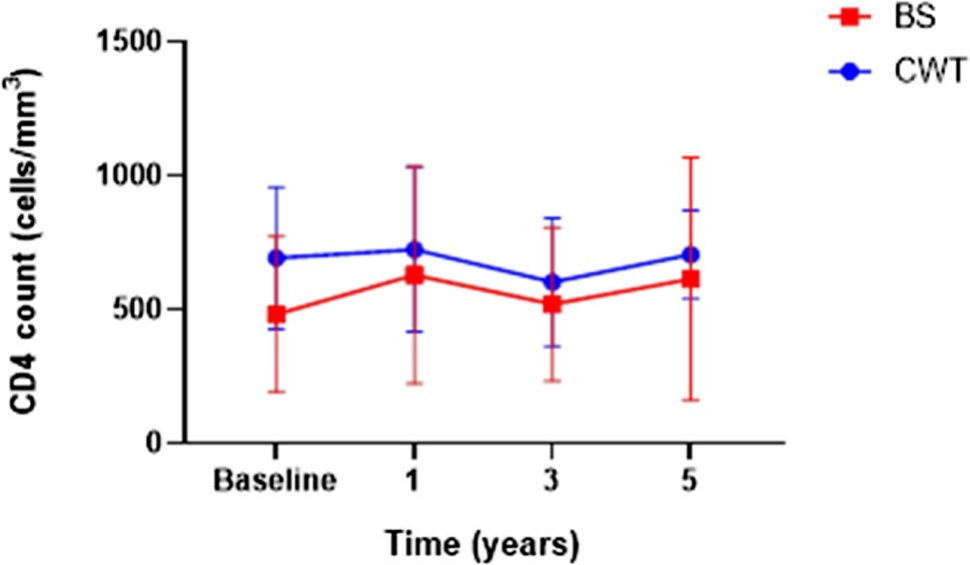
CD4 count at baseline and 1, 3 and 5-year follow-up in patients undergoing Bariatric Surgery and Conservative Weight Therapy. Results shown are plotted as mean and standard error of the mean

The mean CD4 count level was comparable between the 2 groups at baseline, BS 692.6 cells/mm^3^ and CWT 483.2 cells/mm^3^, (*p* = 0.11) at baseline. There was no change in the CD4 count levels in patients undergoing BS or CWT programmes at 1, 3 and 5 years (Fig. 3).

## Discussion

This research article evaluated the impact of bariatric surgery in comparison to conservative weight loss programmes on long-term outcomes in patients with chronic HIV infection and obesity. The primary finding is that bariatric surgery is an effective and safe treatment option for achieving significant and sustained long-term weight loss in this cohort of patients without causing disruption in HIV treatment and outcomes.

In this cohort of patients who underwent BS, the most significant weight loss was achieved during the first year. In terms of the surgical case-mix, there was an equal split between patients undergoing LAGB and stapled procedures, with weight loss being greater with using the latter. This finding is well reported in the published literature base in the general population [18, 19]. In PLWH specifically, similar weight long-term outcomes have been demonstrated in patients undergoing RYGB or LSG in comparison to LAGB mirroring the non-infected individuals. These outcomes may contribute towards informing the selection of BS interventions by healthcare professionals. A systematic literature review conducted by Akbari et al. reported the outcomes of PLWH undergoing RYGB, LSG and mixed cases. Although the overall sample sizes were small in each group, short- and medium-term outcomes suggested that BS is safe and did not impact HIV disease progression [8]. They also mention that there was no difference in HIV-related outcomes between LSG and RYGB. Similar outcomes were reported by Kaip et al. who reported outcomes from a retrospective case series of PLWH undergoing either RYGB or LSG. Both outcomes achieved significant weight loss and maintained virological suppression and CD4 counts at 1 year following surgery [16]. In view of current evidence and changing trends in the selection of bariatric surgery, there is increasing confidence in the efficacy and safety that PLWH can undergo stapled BS interventions that generate superior weight loss in a similar fashion to non-infected individuals.

Although LSG and RYGB have shown more significant weight loss than LAGB, in some cases, they are associated with micronutrient and vitamin deficiencies. These deficiencies are attributed to altered absorption mechanisms resulting from the bypass of gastric and small bowel segments, and in some cases, reduced mixing with digestive enzymes [20]. There are subsequently concerns regarding the uptake, absorption and altered pharmacokinetics and drug availability of highly active anti-retroviral (HAART) therapy in PLWH. Some common HAARTs such as abacavir, dolutegravir, tenofovir and emtricitabine are absorbed in the duodenum and proximal jejunum. Persistent exposure to HAART is of primary importance to prevent disease progression and resistance. Subsequently, anti-retrovirals that require low pH, administration of fatty meals, longer intestinal exposure, and an enterohepatic recirculation for their absorption may be negatively impacted by bariatric surgery procedures [21]. Data from our study demonstrates that there was a stable maintenance of an undetectable viral load and sustained levels CD4 counts in PLWH following surgery, suggesting that the intervention did not adversely impact with HAART treatment. All patients were maintained on consistent HAART therapy with no adverse outcomes or reports of non-compliance. A comprehensive review by Rino et al. evaluated the impact of BS on pharmacokinetics of HAARTs [21]. The key messages were that studies in the intended population such as this current study are crucial as predicting individual pharmacokinetics on the sole basic of drug characteristics are challenging. Generally, HAARTs such as dolutegravir, darunavir and most nucleoside analogue reverse transcriptase inhibitors have been proven successful and safe drug candidates’ post-bariatric surgery. In our study, HAART therapy was overseen by the specialist HIV physicians in our tertiary unit. Most patients were on combination therapy and there were no reports of change to therapy as a sole result of BS.

Although the weight loss achieved was superior from BS than CWT programmes, there was still a tangible reduction in weight generated in the latter group. Considering weight loss in general has proven health and psychosocial benefits, CWT should be considered as management option or alternative to surgery in individuals who do not qualify or wish to undergo BS. Adherence to such programmes are known to be barrier to their success, despite patients being reviewed at frequent intervals by dieticians, physicians and physiotherapists on the CWT, therefore stringent patient selection is crucial if physicians opt to select this intervention for their patients [22]. Using CWT in combination with other weight loss therapies such as medical treatments are likely to achieve much greater success than offering CWT alone. Medical treatments such as semaglutide and liraglutide are FDA approved interventions that show promising results and huge popularity. Such licensed medication have already been backed by NICE for individuals with a BMI greater than 35 kg/m^2^ with at least 1 weight-related comorbidity alongside a reduced-calorie diet and increased physical activity and may have a role in the management of obesity in PLWH [23]. Zino et al. aimed to explore the benefits, safety aspects and pharmacological considerations of semaglutide and liraglutide on PLWH, however this was limited to 2 cases [24]. Early reports on the pharmokinetics of these GLP-1 agonists suggest that they do not adversely interact with HAARTs and they are metabolised using different pathways and enzymes to HAARTs so demonstrate early safety data. The authors highlight that caution should be applied when HAARTs such as atazanavir or rilpivirine is combined with GLP-1 agonists that inhibit gastric acid secretion and hence bioavailability of those HAARTs. Large scale randomised clinical trials such as the SWIFT trial; Semaglutide’s Efficacy in Achieving Weight Loss for Those With HIV, that is currently recruiting will be imperative for provided weight loss and HIV outcomes on the efficacy and safety of semaglutide on weight outcomes as well as immune function and HIV-related parameters [25].

Although the data in this study reflects real life clinical outcomes in the intended population, we report experiences from a single tertiary referral centre with a small sample size which is a limitation of this paper. Larger numbers from multiple centres would allow us to increase numbers which would be particularly useful for the subgroup analysis based on bariatric intervention. In addition, this data was collated from 2012 to 2018. Since then, there have been changing trends in bariatric surgery such as the fall in popularity of LAGB. Half of the patients in the surgical group in this study underwent LAGB placement as a weight loss intervention, however nowadays, its use has significantly declined and been largely superseded by stapled weight loss interventions such as LSG or RYGB. Ongoing data collection and evaluation of PLWH undergoing BS will be important to monitor trends and safety outcome data in these patients.

## Conclusion

Obesity trends are rising in PLWH. Weight loss achieved through any intervention is associated with improved medical and psychosocial outcomes. Bariatric Surgery appears to be a viable and safe medium-term treatment option for individuals with chronic HIV infection, according to this limited dataset. LSG and LRYGB typically result in more substantial weight loss compared to LAGB and CWT. We suggest considering weight management as a critical component of HIV treatment and recommend the timely referral of individuals with severe obesity and chronic HIV infection to a specialized bariatric MDT.

## Data Availability

Data is available upon reasonable request.
